# Long-Term Benefits of Smoking Cessation on Gastroesophageal Reflux Disease and Health-Related Quality of Life

**DOI:** 10.1371/journal.pone.0147860

**Published:** 2016-02-04

**Authors:** Yukie Kohata, Yasuhiro Fujiwara, Takanori Watanabe, Masanori Kobayashi, Yasuhiko Takemoto, Noriko Kamata, Hirokazu Yamagami, Tetsuya Tanigawa, Masatsugu Shiba, Toshio Watanabe, Kazunari Tominaga, Taichi Shuto, Tetsuo Arakawa

**Affiliations:** 1 Department of Gastroenterology, Osaka City University Graduate School of Medicine, Osaka, Japan; 2 Uehonmachi-Watanabe Clinic, Osaka, Japan; 3 Department of Medical Education and General Practice, Osaka City University Graduate School of Medicine, Osaka, Japan; Shiga University of Medical science, JAPAN

## Abstract

**Objective:**

Smoking is associated with gastroesophageal reflux disease (GERD). Varenicline, a nicotinic receptor partial agonist, is used to aid smoking cessation. The purpose of this study was to prospectively examine the long-term benefits of smoking cessation on GERD and health-related quality of life (HR-QOL).

**Methods:**

Patients treated with varenicline were asked to fill out a self-report questionnaire about their smoking habits, gastrointestinal symptoms, and HR-QOL before and 1 year after smoking cessation. The prevalence of GERD, frequency of symptoms, and HR-QOL scores were compared. We also investigated associations between clinical factors and newly-developed GERD.

**Results:**

A total of 141 patients achieved smoking cessation (success group) and 50 did not (failure group) at 1 year after the treatment. The GERD improvement in the success group (43.9%) was significantly higher than that in the failure group (18.2%). The frequency of reflux symptoms significantly decreased only in the success group. There were no significant associations between newly developed GERD and clinical factors including increased body mass index and successful smoking cessation. HR-QOL significantly improved only in the success group.

**Conclusions:**

Smoking cessation improved both GERD and HR-QOL. Smoking cessation should be recommended for GERD patients.

## Introduction

Gastroesophageal reflux disease (GERD) is the most common gastrointestinal (GI) disease encountered in both Western and Asian countries [1, 2]. While not life-threatening, GERD significantly impairs health-related quality of life (HR-QOL) in comparison with that of general adult individuals and patients with other diseases like hypertension and angina pectoris [3]. Lifestyle factors including smoking, being overweight or obese, and consuming late evening meals are commonly identified risk factors for GERD, and the modification of these factors is advocated for GERD management [4]. Systematic reviews have shown the effects of lifestyle modifications on GERD and reflux symptoms, but there was limited efficacy of changing the behavior for reducing GERD symptoms and these studies revealed contradictory results [5, 6].

Several epidemiological studies showed a significant association between smoking and GERD or reflux symptoms [7–10]. Nilsson et al. conducted a case control study of 3153 individuals with severe heartburn or regurgitation and 40210 people without reflux symptoms [9]. They found a significant dose response association between smoking and reflux symptoms. Individuals who smoked >20 cigarettes daily had an increased odds ratio (OR = 1.7) for reflux symptoms compared with non-smokers. The question remains whether smoking cessation affects GERD and reflux symptoms. A large cohort study recently reported that smoking cessation improved severe reflux symptoms only in individuals who had a normal body mass index (BMI) [11]. However, there have been few prospective studies about the impact of smoking cessation on GERD, reflux symptoms, and HR-QOL.

Varenicline (CHANTIX), a nicotinic receptor partial agonist, is used to assist smoking cessation. It is prescribed within Japanese clinics or hospitals when smokers desire to abstain from smoking. The aim of this study was to prospectively examine the long-term clinical benefits of smoking cessation (aided using varenicline) on GERD, reflux symptoms, and HR-QOL.

## Methods

### Subjects

Patients who visited smoking cessation clinics in Osaka City University Hospital and Uehonmachi-Watanabe Clinic of their own volition, and were treated with varenicline between May 2011 and November 2013 were included in this prospective cohort study. Patients who continuously received acid suppressive drugs including proton pump inhibitors (PPI), had peptic ulcer diseases, or had a history of upper GI surgery were excluded. Patients who had undergone smoking cessation therapy for 12 weeks received a survey through the mail 1 year after the treatment. They were asked whether smoking cessation was achieved, and were subsequently divided into success or failure groups. The study was approved by the Osaka City University Ethics Committee, and written informed consent was obtained from all participants, and procedures conformed to the Declaration of Helsinki.

### Smoking Cessation Therapy

Patients initiated varenicline treatment 1 week prior to their intended smoking cessation date. Varenicline was taken once or twice daily after eating according to the following treatment regimen: 0.5 mg once daily from day 1 to day 3, 0.5 mg twice daily from day 4 to day 7, and 1 mg twice daily from day 8 to the end of the treatment. Patients took varenicline for a total of 12 weeks.

### Questionnaire

All participants were asked to complete a questionnaire on their age, gender, height, body weight, smoking habits, alcohol drinking habits, reflux symptoms, and HR-QOL prior to beginning varenicline therapy. BMI was calculated as body weight divided by the square of body height in meters (kg/m^2^). Respondents with a BMI > 25 kg/m^2^ were defined as overweight, while those with a BMI ≥ 30 kg/m^2^ were considered obese. With regard to smoking habits, participants were asked to report the number of cigarettes they smoked per day, the number of years they had been smoking, the amount of tar in their preferred brand of cigarettes, and whether those cigarettes contained menthol. The Brinkman index was calculated by multiplying the number of cigarettes smoked per day by the number of years of smoking. One year after the smoking cessation therapy, the patients were asked to fill out a questionnaire—sent through the mail—regarding their height, body weight, reflux symptoms, HR-QOL, whether they had taken acid-suppressive drugs continuously since completing the therapy, and whether the smoking cessation had remained successful.

### Assessment of GERD

**Fig 1:**
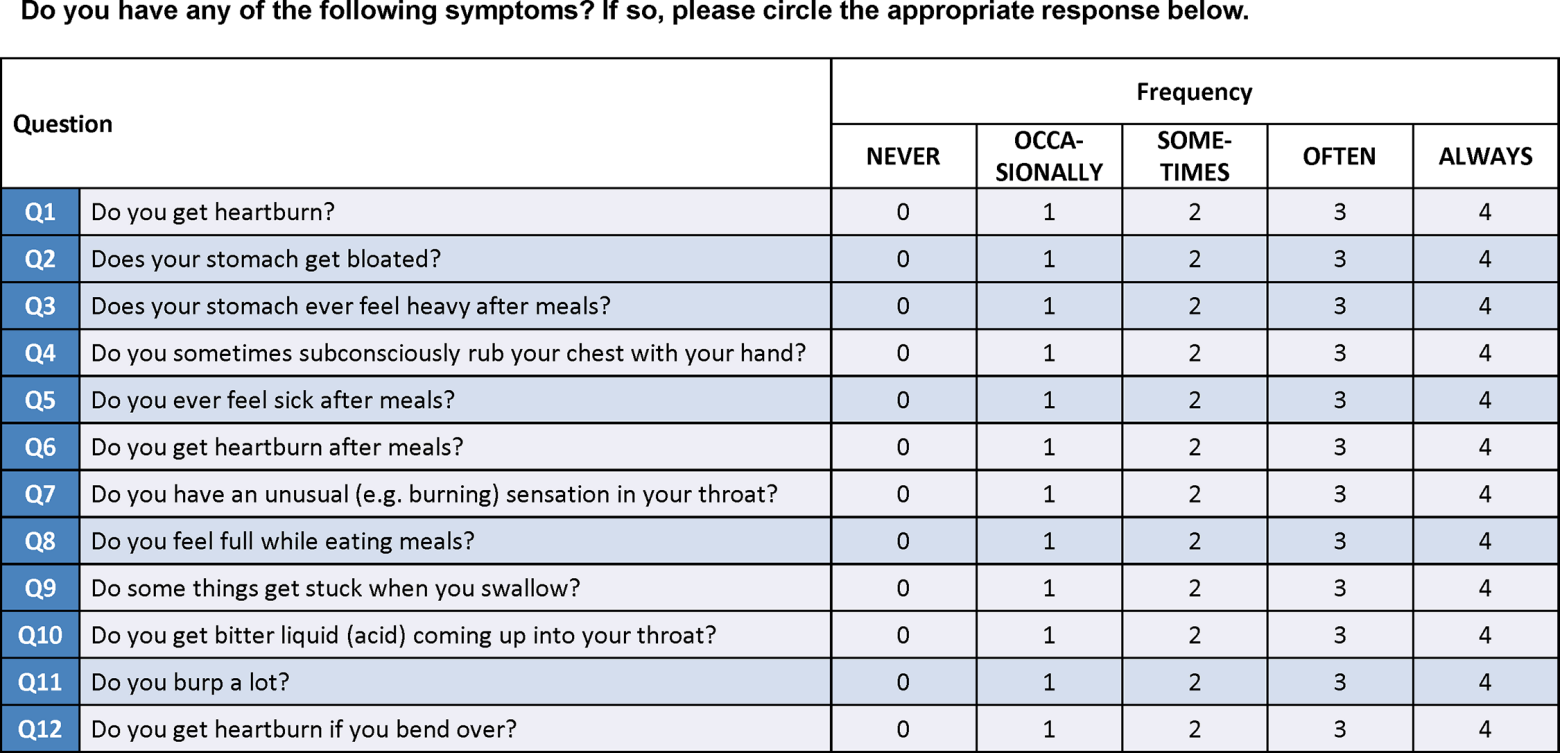
Frequency scale for symptoms of gastroesophageal reflux disease. Questionnaire is consist of 12 questions including 7 (light blue lines) with acid reflux symptoms and 5 (dark blue lines) with dysmotility symptoms.

The frequency scale for the symptoms of GERD (FSSG) was developed in Japan for screening and evaluating the therapeutic response of GERD patients (Fig 1). The FSSG questionnaire consists of 12 questions, 7 (Q1, Q4, Q6, Q7, Q9, Q10, and Q12) about acid reflux-related symptoms (reflux score) and 5 (Q2, Q3, Q5, Q8, and Q11) about dysmotility (dyspeptic) symptoms (dysmotility score). Patients assigned each question 0 (never), 1 (occasionally), 2 (sometimes), 3 (often), and 4 (always) points. Patients with a total score of ≥8 on the FSSG were diagnosed with GERD, with a sensitivity of 62%, a specificity of 59%, and an accuracy of 60% [12]. Patients with a total score of <8 on the FSSG 1 year after the varenicline treatment were considered to have improved.

### Assessment of HR-QOL

HR-QOL was assessed using the Japanese version of the 8-item Short-Form Health Survey (SF-8) [13], a questionnaire derived from the 36-item Short-Form Health Survey (SF-36) developed to estimate HR-QOL based on the scores from the following 8 domains and 2 summaries: physical functioning, physical role, bodily pain, general health perception, vitality, social functioning, emotional role, mental health, and the physical and mental component summaries. The physical functioning, role physical, and bodily pain scores contribute most to the physical component summary, while the mental component summary is mainly derived from the mental health and role emotional scores. The remaining three domains (vitality, general health perception, and social functioning) correlate with both physical and emotional components [13–15]. The physical and mental component summary scores are calculated based on the Japanese manual for the SF-8. An average score of 50 is typical for the Japanese general population. A lower score indicates a worse HR-QOL [13].

### Statistical Analysis

Data were expressed as mean and standard deviation (SD), frequency, or OR with 95% confidence intervals (CI). Factors including age, gender, BMI, alcohol drinking habits (drinker or non-drinker), and smoking habits were compared between patients within the smoking cessation success and failure groups using the student’s t-test or chi square test. The prevalence of GERD symptoms, frequency of symptoms (FSSG score), BMI changes, and HR-QOL score before and 1 year after smoking cessation were compared between patients within the success and failure groups using the chi square test or Wilcoxon signed rank test. We also investigated relationships between clinical characteristics such as age, gender, BMI increase, successful smoking cessation, and alcohol drinking habits and newly-developed GERD 1 year after smoking cessation in patients who had not had GERD at the first visit. A forward stepwise multiple logistic regression model was created to assess independent associations between risk factors and newly-developed GERD. P values <0.05 were considered significant. Statistical analyses were performed using SPSS version 16.0 J (SPSS Japan, Inc., Tokyo, Japan).

## Results

### Study Subjects

**Fig 2:**
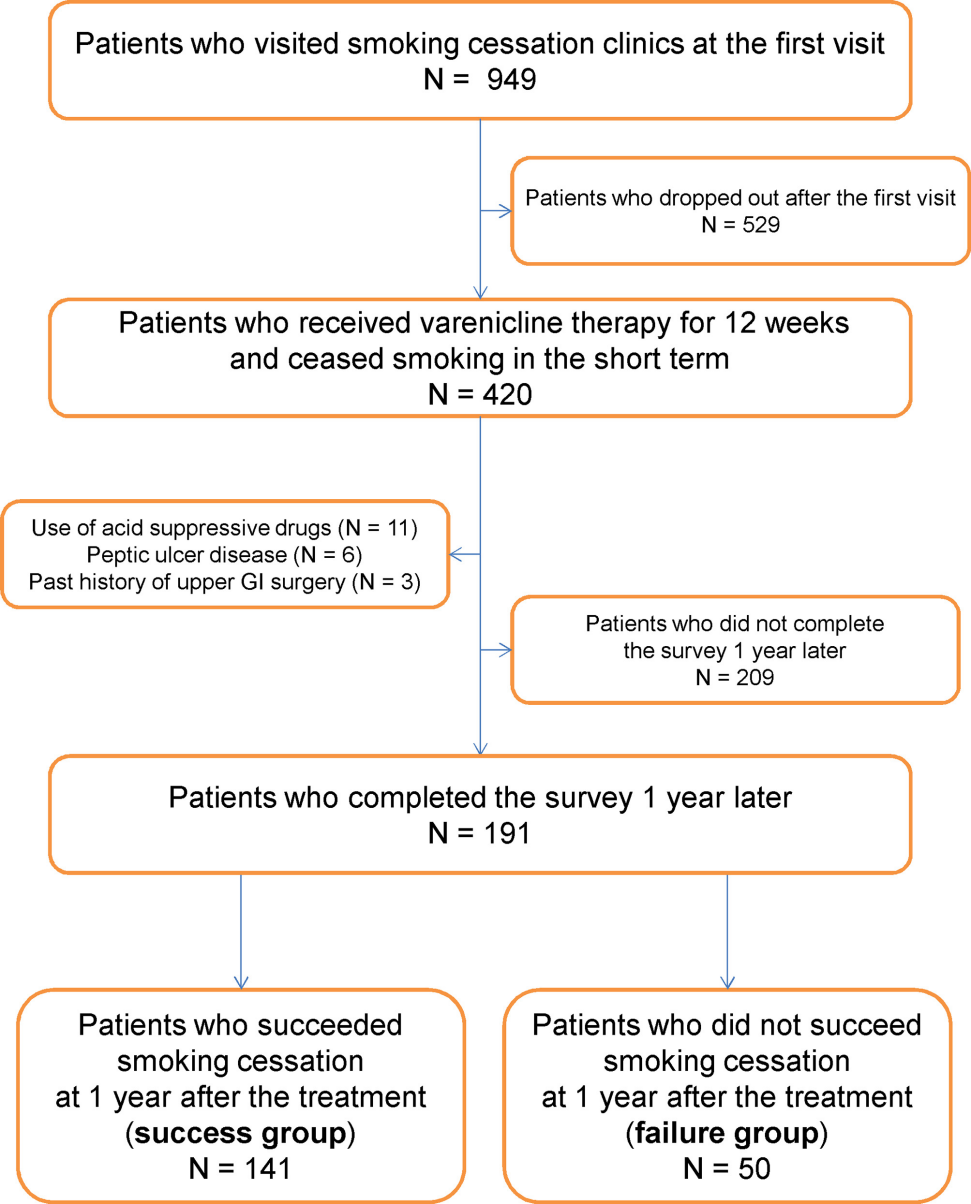
Flow chart of study participants. Among 949 patients who initially visited smoking cessation clinics, 420 underwent smoking cessation therapy using varenicline for 12 weeks; all these patients ceased smoking in the short term. Twenty patients who used acid suppressive drugs, had peptic ulcer disease, or had a history of upper GI surgery were excluded, and 209 patients did not complete the survey 1 year later. Of 191 patients who completed the survey 1 year after attempted smoking cessation, 141 patients achieved smoking cessation (success group) and 50 did not (failure group).

A total of 949 patients who visited smoking cessation clinics during the study period were initially enrolled in the study, but 529 patients did not remain in the study after the first visit. Of the remaining 420 patients who had received varenicline for 12 weeks and had ceased smoking short-term, 20 were excluded from the study because of acid-suppressive drug use (n = 11), peptic ulcer disease (n = 6), or a history of surgery in the upper GI tract (n = 3). An additional 209 patients did not complete the survey 1 year later. A total of 191 patients were included in the final analysis, 141 patients who succeeded smoking cessation (success group) and 50 who did not (failure group) (Fig 2). No patients received continuously acid suppressive drugs during the research period. Table 1 shows the baseline clinical characteristics of the study subjects at the first visit before smoking cessation. There were no significant differences between the success and failure groups in terms of age, gender, BMI, obesity, or alcohol drinking habits. There were also no significant differences between the two groups with regard to smoking habits including the number of cigarettes per day, number of years smoking, Brinkman index, and amount of tar or presence of menthol in the cigarettes. There was no difference in the prevalence of GERD at baseline between the success group (36.2%) and the failure group (44.0%) (p = 0.33). To determine whether there were any observable sources of bias, we compared the baseline clinical characteristics of the 191 patients in the final analysis with those of the 209 patients who had not responded to the follow-up questionnaire distributed 1 year after the treatment. There were no significant differences between the two groups in terms of baseline data (mean age: 46.8 ± 13.5 years in patients with follow-up *versus* 44.9 ± 12.5 years in patients without follow-up [p = 0.13]; percentage of men: 59.7% in patients with follow-up *versus* 65.5% in patients without follow-up [p = 0.22]; BMI: 22.7 ± 3.6 kg/m^2^ in patients with follow-up *versus* 22.9 ± 3.3 kg/m^2^ in patients without follow-up [p = 0.46]; prevalence of overweightness: 24.1% in patients with follow-up *versus* 27.8% in patients without follow-up [p = 0.40]; prevalence of obesity: 3.7% in patients with follow-up *versus* 1.9% in patients without follow-up [p = 0.28]; proportion of alcohol drinkers: 52.4% in patients with follow-up *versus* 58.4% in patients without follow-up [p = 0.22]; number of cigarettes per day: 21.2 ± 12.0 in patients with follow-up *versus* 23.1 ± 9.9 in patients without follow-up [p = 0.09]; number of years of smoking: 24.6 ± 13.1 years in patients with follow-up *versus* 23.0 ± 11.8 years in patients without follow-up [p = 0.21]; Brinkman index: 517.8 ± 403.3 in patients with follow-up *versus* 542.3 ± 377.1 in patients without follow-up [p = 0.53]; prevalence of GERD: 38.2% in patients with follow-up *versus* 39.7% in patients without follow-up [p = 0.75]).

**Table 1:** Clinical characteristics of the study subjects at baseline.

	Success group (N = 141)	Failure group (N = 50)	P-value
Age (yrs)	47.0 ± 13.9	46.4 ± 12.4	0.80
Gender (male/female)	82 / 59	32 / 18	0.46
BMI (kg/m^2^)	22.6 ± 3.7	22.9 ± 3.5	0.60
Prevalence of overweightness (%)	21.9	28.0	0.38
Prevalence of obesity (%)	3.5	4.0	0.59
Alcohol drinker (%)	52.5	52.0	0.95
Smoking habits			
Number of cigarettes per day	20.4 ± 9.7	23.4 ± 16.7	0.13
Number of years of smoking (yrs)	25.0 ± 13.1	23.2 ± 13.2	0.38
Brinkman index	514.2 ± 370.9	528.0 ± 487.3	0.83
Tar per cigarette (mg)	5.3 ± 4.0	5.2 ± 4.2	0.92
Menthol (%)	12.8	24.0	0.06
Prevalence of GERD (%)	36.2	44.0	0.33

Data presented as mean ± standard deviation (SD) or frequency (%). BMI, body mass index; GERD, gastroesophageal reflux disease.

### Changes in BMI

The BMI of the patients within the success group significantly increased from 22.5 ± 3.6 kg/m^2^ at baseline to 23.3 ± 3.5 kg/m^2^ (p<0.01) at 1 year after the treatment, while the BMI of patients within the failure group did not change (22.9 ± 3.4 kg/m^2^ at baseline versus 22.8 ± 3.4 kg/m^2^ at 1 year after therapy, p = 0.62). The prevalence of obesity did not change in either the success group (22% at baseline versus 24% at 1 year after the treatment, p = 0.67) or the failure group (28% at baseline versus 26% at 1 year after the treatment, p = 0.82).

### Changes in Prevalence of GERD and Frequency of Reflux Symptoms

**Fig 3:**
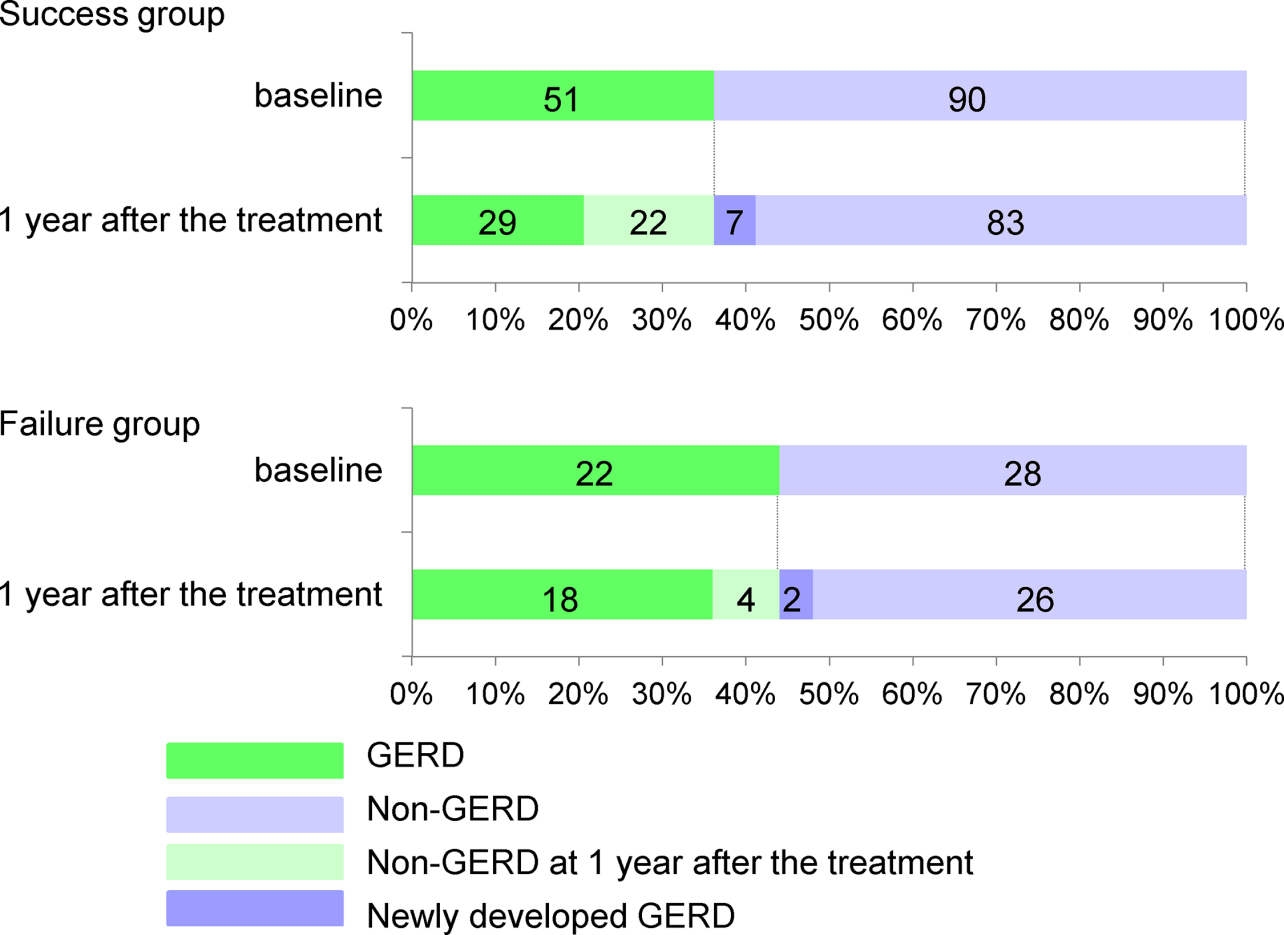
Prevalence of GERD at baseline and 1 year after attempted smoking cessation. The number of patients that experienced improvement in GERD was significantly higher in the success group (43.9%) than in the failure group (18.2%). Seven (7.8%) of the patients within the success group and 2 (7.1%) of the 28 patients within the failure group newly developed GERD at 1 year after the treatment.

**Fig 4:**
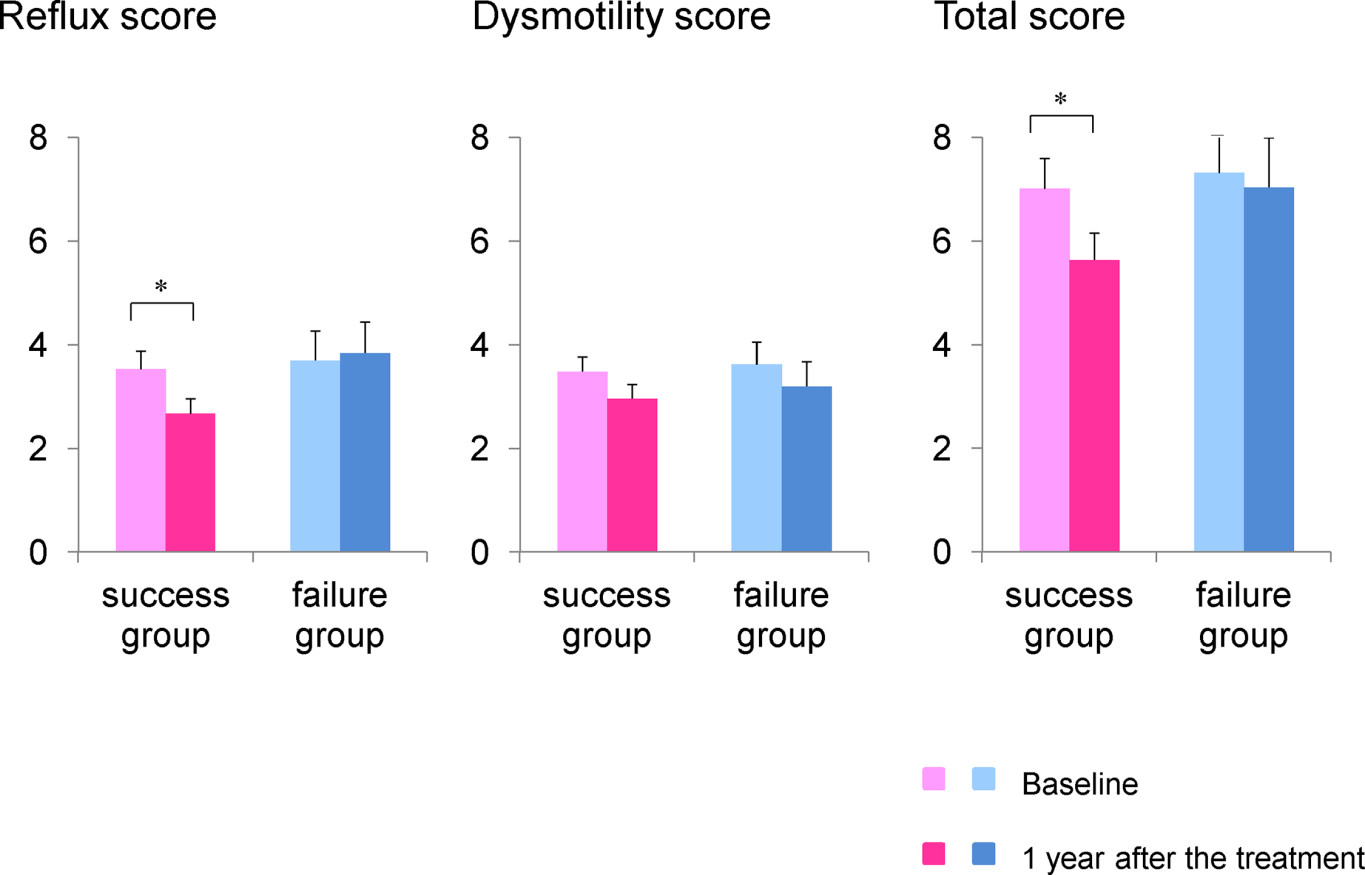
Changes in FSSG score among all subjects. The reflux and total scores significantly decreased 1 year after smoking cessation in the success group, but not in the failure group. *p<0.05 versus baseline.

Fig 3 shows the prevalence of GERD at the first visit and 1 year after attempted smoking cessation in each group. The number of patients that experienced improvement in GERD in the success group was significantly higher than in the failure group (43.9% in the success group versus 18.2% in the failure group, p<0.05). The total FSSG and reflux scores in the success group significantly decreased 1 year after smoking cessation, while the dysmotility score did not change. There were no significant differences in the total score or sub-scores in the failure group (Fig 4).

Nine (7.6%) of the 118 patients without GERD at baseline newly developed GERD 1 year after smoking cessation therapy, which included 7 (7.8%) of the 90 patients within the success group and 2 (7.1%) of the 28 patients within the failure group. By multivariate analysis there was no significant association between newly-developed GERD and clinical factors including age, gender, BMI increase, successful smoking cessation, or alcohol drinking habits (Table 2).

**Table 2:** Factors associated with new development of gastroesophageal reflux disease.

	Crude OR	Multiple-adjusted OR
Age (per 1 year)	0.96 (0.91–1.12)	0.94 (0.89–1.01)
Male gender	0.26 (0.06–1.09)	0.39 (0.08–1.96)
BMI increase (per kg/m^2^)	0.73 (0.17–3.09)	0.54 (0.09–3.14)
Successful smoking cessation	1.10 (0.21–5.61)	1.25 (0.20–7.70)
Alcohol drinker	0.12 (0.01–0.98)	0.12 (0.01–1.11)

Data presented as crude odds ratio (OR) (95% confidence interval, CI) and multiple-adjusted ORs (95% CI) after adjustment for age, gender, body mass index (BMI), successful smoking cessation, and alcohol drinker.

### Changes in HR-QOL

**Fig 5:**
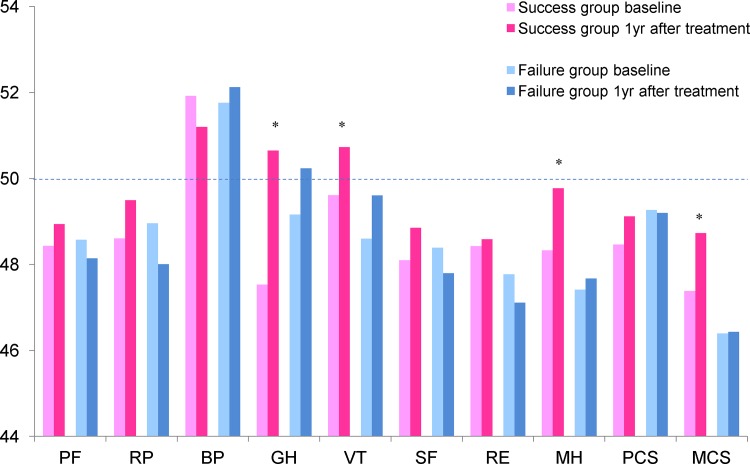
HR-QOL (SF-8 survey score) before and after smoking cessation. General health (GH), vitality (VT), and mental health (MH) significantly improved after smoking cessation in the success group, whereas there were no significant changes in the failure group. PF, physical functioning; RO, role physical; BP, bodily pain; SF, social functioning; RE, role emotional; PCS, physical component summary; MCS, mental component summary. *p<0.05 versus baseline.

HR-QOL was evaluated using the SF-8 survey before and after attempted smoking cessation in each group. Several SF-8 domains significantly improved after smoking cessation in the success group, including general health, vitality, and mental health, whereas there were no significant changes in any domains in the failure group. The mental component summary score also improved only in the success group, but the physical component summary score did not (Fig 5).

## Discussion

To our knowledge, this is the first study which prospectively investigated the long-term effects of varenicline-induced smoking cessation on GERD, reflux symptoms, and HR-QOL. The present study showed that successful smoking cessation by varenicline associated with decreased prevalence of GERD, reduced frequency of reflux symptoms, and improved HR-QOL. Approximately 7% of patients who had not previously had GERD newly developed GERD after varenicline therapy, but there was no association with age, gender, BMI increase, successful smoking cessation, or alcohol drinking habits.

Recently, the HUNT study reported that quitting smoking improved reflux symptoms, but only in individuals of normal weight [11]. Although this study was a large, prospective, population-based cohort study conducted from 1995–1997 and 2000–2009, it did not detail whether improvement of reflux symptoms occurred before or after smoking cessation, and only three simple response alternatives (none, minor, severe) were used to describe reflux symptoms. In contrast, our study utilized a validated questionnaire for GERD symptoms and defined an exact time period after smoking cessation for analysis of all endpoints.

The HUNT study also reported risk factors for new onset of reflux symptoms[16]. They identified that male sex and higher education were negatively associated with new-onset reflux symptoms, while an increase in BMI, and previous or current smoking were positively associated. Smoking cessation was associated with a new-onset of reflux symptoms among patients with increased BMI upon quitting. In our study, sex, smoking cessation, and BMI increases did not associate with new development of GERD among smokers who visited clinics for smoking cessation. The exact reasons for discrepancies between the HUNT study and our study are unknown, but the number of subjects, observation period (1 year versus approximately 5 years), and ethnicity of patients might be associated. In particular, obesity is rare in Japan, and the mean baseline BMI of our study subjects was 22.6 kg/m^2^, while that of the HUNT study subjects was approximately 26.0 kg/m^2^.

Early studies demonstrated that smoking reduced lower esophageal sphincter (LES) pressure and prolonged acid clearance through a decrease in saliva bicarbonate secretion [17–19]. Kahrilas and Gupta showed that smokers exhibited lower LES pressures compared with non-smokers, and smoking increased acid reflux events through an abrupt increase in intra-abdominal pressure during coughing or deep inspiration [20]. Two studies demonstrated that a short period (24hr) of abstaining from smoking did not influence esophageal acid exposure time in both subjects with and without reflux symptoms [21, 22]. However, Kadakia et al. showed a significant reduction in total acid reflux 48hrs after smoking cessation [23]. Apart from these studies, our study showed the long-term effects of smoking cessation on GERD and reflux symptoms. Improvement of GERD in our study might be due to normalization of LES pressure and saliva bicarbonate secretion after smoking cessation.

Since varenicline is associated with several GI side effects such as nausea, epigastralgia, and indigestion, we could not judge the exact effect of smoking cessation on GERD symptoms during or just after the varenicline therapy. Instead, we conducted the survey 1 year after completion of varenicline treatment since these side effects subside before 1 year after treatment.

Our study showed successful smoking cessation improved HR-QOL. Several other studies showed that successful smoking cessation resulted in improved well-being using subjective methods of evaluation [24, 25]. Kinoshita et al. reported that lifestyle modification, including smoking cessation together with PPI therapy, significantly improved HR-QOL compared with PPI therapy without lifestyle modification [26]. A decrease in the prevalence of GERD and frequency of reflux symptoms by smoking cessation might, in part, explain the improved HR-QOL.

There are some limitations of this study. First, the number of subjects was relatively small, especially considering that approximately half of the patients did not complete the survey 1 year after smoking cessation. However, we posit that there was no bias, as the group that provided the follow-up questionnaire did not differ significantly in terms of baseline characteristics from the group that did not. Second, GERD was diagnosed based on a specific questionnaire but endoscopy or esophageal pH-metry was not utilized to confirm the presence of the disease. Third, it is possible that some patients had GERD with relatively mild symptoms because they did not seek medications such as PPI during the study period. Fourth, we assessed smoking status 1 year after the treatment by using a self-report questionnaire alone. Future studies that employ biochemical verification by measuring exhaled carbon monoxide, cotinine, or thiocyanate levels should be conducted. Finally, we found no significant association between BMI increase and new development of GERD. Recent studies emphasized that waist circumference and visceral obesity are more important risk factors for GERD than BMI (27). Future studies including changes in waist size rather than just looking at BMI would be useful.

In summary, smoking cessation reduced the prevalence of GERD, reduced the severity of reflux symptoms, and improved HR-QOL. However, neither success nor failure of smoking cessation influenced new development of GERD during the first year after attempted smoking cessation. Smoking cessation should be recommended for GERD patients.

## Supporting Information

S1 Dataset Final analysis data of the study subjects. (XLSX)
