# Correction: Long-Term Benefits of Smoking Cessation on Gastroesophageal Reflux Disease and Health-Related Quality of Life

**DOI:** 10.1371/journal.pone.0150554

**Published:** 2016-03-01

**Authors:** Yukie Kohata, Yasuhiro Fujiwara, Takanori Watanabe, Masanori Kobayashi, Yasuhiko Takemoto, Noriko Kamata, Hirokazu Yamagami, Tetsuya Tanigawa, Masatsugu Shiba, Toshio Watanabe, Kazunari Tominaga, Taichi Shuto, Tetsuo Arakawa

There is an error in the Results subsection of the Abstract as well as third paragraph of the Results section. In both of these instances, 43.9% should be 43.1%.

There is an error in the caption of Fig 3. The authors have provided a corrected caption for Fig 3 here.

**Fig 3:**
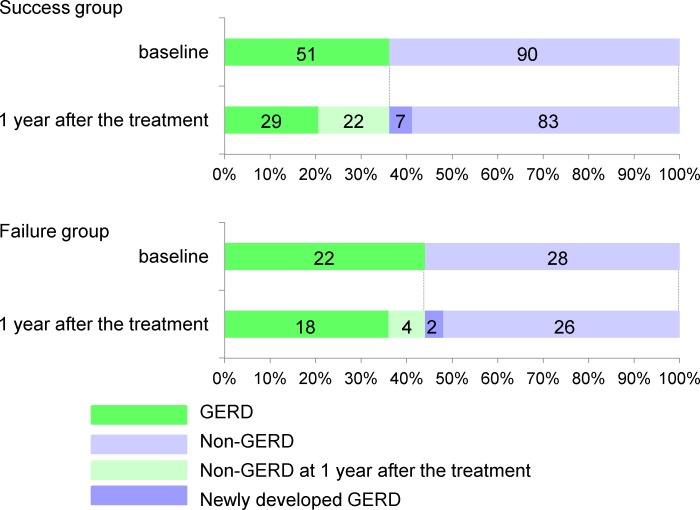
Prevalence of GERD at baseline and 1 year after attempted smoking cessation. The number of patients that experienced improvement in GERD was significantly higher in the success group (43.1%) than in the failure group (18.2%). Seven (7.8%) of the patients within the success group and 2 (7.1%) of the 28 patients within the failure group newly developed GERD at 1 year after the treatment.
